# Commercial Divers’ Subjective Evaluation of Saturation

**DOI:** 10.3389/fpsyg.2018.02774

**Published:** 2019-01-11

**Authors:** Jean Pierre Imbert, Costantino Balestra, Fatima Zohra Kiboub, Øyvind Loennechen, Ingrid Eftedal

**Affiliations:** ^1^Divetech, Biot, France; ^2^Environmental and Occupational Physiology Laboratory, Haute Ecole Bruxelles-Brabant HE2B, Brussels, Belgium; ^3^DAN Europe Research, Brussels, Belgium; ^4^Department of Circulation and Medical Imaging, Faculty of Medicine and Health Sciences, Norwegian University of Science and Technology, Trondheim, Norway; ^5^TechnipFMC, Stavanger, Norway; ^6^Faculty of Nursing and Health Sciences, Nord University, Bodø, Norway

**Keywords:** saturation diving, diving fatigue, headache, hemoglobin, long term health effects, relative hypoxia

## Abstract

Commercial saturation diving involves divers living and working in an enclosed atmosphere with elevated partial pressure of oxygen (ppO_2_) for weeks. The divers must acclimatize to these conditions during compression, and for up to 28 days until decompression is completed. During decompression, the ppO_2_ and ambient pressure are gradually decreased; then the divers must acclimatize again to breathing normal air in atmospheric pressure when they arrive at surface. We investigated 51 saturation divers’ subjective evaluation of the saturation and post-decompression phase via questionnaires and individual interviews. The questions were about decompression headaches and fatigue; and time before recovering to a pre-saturation state. Twenty-two (44%) of the divers who responded declared having headaches; near surface (44%) or after surfacing (56%). 71% reported post-saturation fatigue after their last saturation, 82% of them described it as typical and systematic after each saturation. Recovery was reported to normally take from 1 to 10 days. The fatigue and headaches observed are compatible with divers’ acclimatization to the changes in ppO_2_ levels during saturation and decompression. They appear to be reversible post- decompression.

## Introduction

Commercial saturation diving in the North Sea started with the emergence of the offshore oil and gas industry in 1969. The North Sea has depths from 100 to 180 meters of sea water (msw) that became the standard range of manned underwater operations. In 1980, the interest shifted to deeper diving in Norway and in Brazil. A series of contracts was awarded in Norway for validating diver interventions to 300–350 msw. During this period, outstanding developments were conducted at the Norwegian Underwater Technology Centre (NUTEC) in Bergen and several deep saturation dives were performed in the Norwegian fjords (Hope et al., 2005a).

However, at the turn of the 90s, the Norwegian media raised the issue of potential long-term health effects of deep diving. This became a national debate and in 1993, the Godøysund conference concluded that standard saturation diving in the Norwegian continental shelf should be limited. Today, NORSOK-U100 standards (Standards Norway, 2014) that regulates manned underwater operations in Norway limits standard operations to 180 msw.

The limitation of diving depths in Norway was not the end of deep diving. The expertise was transferred from Norway to Brazil by companies like Comex that were operating internationally. In Brazil, deep diving to 200–240 msw became a routine (Hope et al., 2005b). The key factors for preserving divers’ health identified during the Brazilian operations were: (a) selection on experience, (b) selection on fitness, and (c) progressive adaptation to increasing depths (Vivaqua, 2017).

A second conference held in Norway in 2005 revisited the 1993 Godøysund conclusions. This conference first accounted for the evolution of diving procedures and the success of Brazilian operations. It also permitted separating the issues of the potential neurological effects due to long exposures to elevated partial pressure of oxygen (ppO_2_), the effect of circulating venous gas on the lung function (Thorsen et al., 1995), and oxygen effects. Oxygen effects were further split into oxygen pulmonary toxicity (Thorsen et al., 1990, 1993) and changes in blood hemoglobin concentration (Hofso et al., 2005). During the conference, the results of the Examination of Long Term Health Impact of diving (ELTHI) study were presented (Murray et al., 2004). The ELTHI study failed to detect any long-term health effects except for welder divers.

**FIGURE 1:**
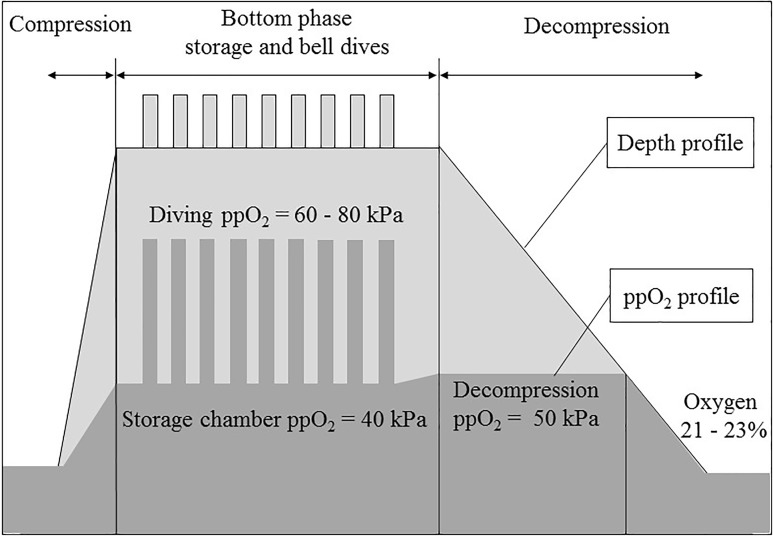
A typical saturation profile (compression, storage and bell dives, decompression at constant partial pressure of oxygen -ppO_2_- and at constant oxygen percentage) and the corresponding ppO_2_ profile.

Saturation procedures use higher than normal levels of oxygen in the breathing gas, as illustrated in Figure 1 for a typical saturation profile. We therefore postulated that some of the symptoms reported during and after decompression from saturation were related to relative hypoxia when the diver returns to surface; breathing normobaric air with a ppO_2_ of 21 kPa. The rationale for this relative hypoxia was based on the succession of two oxygen exposures. In the first step, during saturation, the divers become acclimatized to elevated levels of oxygen. This is supported by observations of a reduction in red blood cell counts. Drops in hematocrit and hemoglobin concentrations have also been measured in divers after a period of saturation, although the values remained within normal range (Thorsen et al., 2001; Deb et al., 2017; Kiboub et al., 2018a,b).

In a second step, by the end of the decompression, the ppO_2_ decreases and the oxygen content in the chamber breathing gas must be kept below 23% to prevent the risk of fire. Depending on the diving procedures, this happens at 12–13 msw and lasts for 16–21 h until the chamber reaches surface pressure. At the end of the decompression, this drop of breathing gas ppO_2_ is perceived by the body as hypoxia (De Bels et al., 2012; Kiboub et al., 2018a).

Our objective in this study was to assess signs and symptoms of hypoxia in saturation divers surfacing from decompression in the North Sea, by implementing an evaluation questionnaire.

## Materials and Methods

### Ethics Statement

This study involved professional divers working in the North Sea, with TechnipFMC as a diving contractor. The study protocol was approved by the Academic Ethical Committee of Brussels (B20-2009-039) and TechnipFMC Diving and Health services in Norway and United Kingdom divisions. Data collection was conducted according to the Declaration of Helsinki principles for ethical human experimentation (World Medical Association, 2013). All participants agreed in writing on individual consents before inclusion in the study. Data collected from the questionnaires were included into an Excel database. The database complied with the 2018 European general data protection regulations; it was fully anonymized, and the anonymization was irreversible.

### Worksite

The monitoring sessions were conducted onboard the Diving Support Vessel (DSV) Deep Arctic between 2015 and 2016. The study covered two operations; one in the Norwegian sector at 110 msw storage depth (121 msw working depth) and the other in the United Kingdom sector at 136 msw storage depth (155 msw working depth). In the two sectors, the divers used the same breathing gasses and equipment and performed the same type of work (well-intervention). However, the saturation procedures differed slightly because of local regulations.

### Study Group

The study group consisted of male certified commercial saturation divers. All divers who were cleared for diving by the hyperbaric nurse during the pre-dive medical examination, were eligible for participation. The divers were organized in teams of three men. Each team was involved in one excursion per day during their 12 h-shift. The divers’ shift time was fixed over the saturation period, and the shifts were distributed over the day as the vessel operated a two-bells system for 24 h coverage. Bell run time was limited to 8 h and diver’s in-water time to 6 h; with a 30-min restitution break inside the bell in the middle of the in-water time. Table 1 describes the study group demographics, total and saturation diving experience and body-mass index (BMI).

**Table 1:** Subjects (*n* = 51) biometric information and experience expressed in mean (range).

	Age	Total diving	Saturation diving	BMI
	[years]	[years]	[years]	[kg/m^2^]
Mean (range)	46.1 (31 to 61)	21.8 (6 to 42)	15.9 (2 to 39)	26.5 (21.0 to 32.3)


### Saturation Procedures

Saturation was conducted using heliox (helium and oxygen mixture) as breathing gas. Two saturation procedures were used, NORSOK U-100 saturation procedures (Standards Norway, 2014) in Norway, and the TechnipFMC standard saturation procedures in the United Kingdom; which only differ in the final decompression.

During compression, the chamber ppO_2_ was maintained between 21 and 45 kPa. The divers were pressurized to storage depth at a rate of 1 m/min. At storage depth, the chamber ppO_2_ was kept between 38 and 42 kPa. During the excursions, the breathing gas ppO_2_ varied from 60 to 80 kPa. During decompression, the chamber ppO_2_ was kept between 46 and 50 kPa in Norway and between 48 and 52 kPa in the United Kingdom (difference of 2 kPa, or 4%). Close to surface, the chamber oxygen percentage was kept between 21 and 23% in both procedures.

The decompression durations were 5 days and 4 h in the Norwegian project and 5 days 13 h in the United Kingdom project. The decompression durations differed by 9 h (5%). The maximum saturation durations are limited to 14-days bottom time in Norway and 28-days total time in the United Kingdom. The actual saturation durations varied according to the operational needs.

### Questionnaire

The study was part of a larger project of divers’ monitoring, aiming at collecting information on divers’ high pressure nervous syndrome (HPNS) symptoms, fatigue, heat and cold exposure, stress, sleep, and hydration. The study was based on a questionnaire focusing on the diver’s subjective evaluation of oxygen acclimatization:

- Diver’s experience of headaches during or after decompression,
- Diver’s subjective evaluation of post-saturation fatigue,
- Time required to return to normal pre-saturation state regarding the factors above,
- Diver’s strategy to cope with return to normal life.

The questionnaire included boxes to tick-off and, when relevant, a visual scale using a line of 10 cm (0 to 10) allowing a continuous evaluation. The divers were asked to complete the questionnaire within 12 h after surfacing from decompression. Some then went into structured interviews with the investigator in a separate room in the vessel’s hospital, one at a time. The objective was to provide explanations if needed and ensure that all the questions were answered. The interviewer did not influence or change the divers’ answers at any stage.

### Transthoracic Venous Gas Bubble Detection

Transthoracic ultrasound echography and Doppler examinations for circulating venous gas bubble detection were conducted within 2 h of the end of decompression. A Mindray M7 echocardiograph (Mindray, Shenzhen, China), equipped with a 2.5 MHz linear array transducer was used. Each diver was at rest on a bed in supine position for 3 min, before his heart was examined in an apical four chamber view as described in Bulwer and Rivero (2010). After the first examination, the subject performed a series of three squats prior to a second examination to detect potential bubbles released after the effort. Venous bubbles were also monitored using the Doppler. Several video sequences of 250 frames were registered for each diver, and used for the bubbles count. The Eftedal-Brubakk bubbles grading system was to be referred to in the eventual presence of bubbles (Eftedal and Brubakk, 1997).

### Statistical Analysis

Different statistical tests were adopted depending on the nature of the data. Two-sided Fisher exact tests were applied with categorical data. Pearson’s test was conducted to examine correlations between age and recovery time. *P*-values < 0.05 were considered significant.

## Results

The diving operations were concluded without any incidents. A total of 51 divers were invited to participate in the study, and all of them accepted and answered the questionnaire (*n* = 29 in the Norwegian sector, and *n* = 22 in the United Kingdom sector). The average saturation duration for the divers involved in the study was 19.7 ± 6.5 days. No bubbles were detected after saturation in any of the divers, either in the ultrasound images or by Doppler. The questions’ response rates and results are described in Tables 2, 3. There was no correlation between age and recovery time (correlation coefficient = 0.023).

**Table 2:** Questions related to headaches.

Questions	Participant’s number	Scores	Response percentage (%)
Q1: During this last decompression, have you experienced headaches?	34	Yes = 11 No = 23	32 68
Q2: During this last decompression, if you had headache, when did the symptoms declare?	11	Near surface = 6 After surfacing = 5	55 45
Q3: During this last decompression, if you had headache, grade its severity on a scale from 1 (light) to 10 (severe)	10	4 (grades 1 to 2) 6 (grades 6 to 9)	40 60
Q4: Usually, do you experience headache during or after decompression?	34	Never = 14 Sometimes = 10 Often = 5 Always = 5 Sometimes + often = 15	41 29 15 15 44
Q5: Usually, if you had headache, when do the symptoms declare?	16	Near surface = 9 After surfacing = 7	44 56
Q6: Usually, if you had headache, how long does the headache last?	13	Few hours = 3 Half a day = 3 One day = 4 More than 1 day = 3	23 23 31 23
Q7: When back home after a saturation, do people around you say you look pale?	20	Yes = 19 No = 1	95 5


**Table 3:** Questions related to post-saturation fatigue.

Questions	Participant’s number	Scores	Response percentage (%)
Q8: After this last saturation, have you experienced fatigue?	24	Yes = 17 No = 7	71 29
Q9: After this last saturation, if you experienced fatigue, grade its severity on a scale from 1 (light) to 10 (severe)	17	(Mean ± SD) 3.4 ± 1.92	100
Q10: Usually, after a saturation, do you experience fatigue?	22	Yes = 18 No = 4	82 18
Q11: Usually, if you experienced fatigue, is it physical or mental?	19	Physical = 17 Mental = 2	89 11
Q12: Usually, after a saturation, grade your well-being or mood on a scale from 1 (bad) to 5 (normal) and 10 (excellent)	22	(Mean ± SD) 6.18 ± 1.56	100
Q13: Usually, after a saturation, grade your alertness on a scale from 1 (bad) to 5 (normal) and 10 (excellent)	22	(Mean ± SD) 6.23 ± 1.91	100
Q14: Usually, after a saturation, how many days does it take to return to normal?	26	(Mean ± SD) 4.31 ± 2.92	51


## Discussion

Although the storage and diving depths were different in the Norwegian and United Kingdom sectors, the same breathing gasses and equipment were used; and the work scope was similar. The decompression durations and chamber ppO_2_ were comparable; and no bubbles were detected, suggesting that the decompression stresses in the two sectors were also similar. Therefore, the data for the two saturation procedures in the Norwegian and United Kingdom sectors were merged in further discussion.

### Oxygen Levels in Saturation

The use of oxygen in diving is a trade-off between positive and negative effects. Saturation procedures use elevated oxygen content. The principle is that a high ppO_2_ in the breathing gas mixture increases the inert gas gradient and accelerates its elimination during decompression. The disadvantage is the negative effect of elevated oxygen levels on the pulmonary function, and its potential toxicity on the central nervous system (Davis et al., 1983; Manning, 2016).

The ppO_2_ values used in commercial saturation diving have been empirically set. For instance, the chamber ppO_2_ at storage depth, which is currently around 40 kPa, came after a chamber with an external regeneration system had a pipe rupture. The chamber dropped from 150 to 70 msw before the chamber operators could close the skin valves. At the time, it was though that if a chamber could drop half its depth, then the chamber ppO_2_ should be twice the normal value for the divers to avoid hypoxia. And it has remained thus since.

#### Post-saturation Headaches

Headaches were frequently reported by the end of decompression. The headache score was 32% (question Q1). When combining the groups “often” and “sometimes” in question Q4, the score became 44%, no difference was found between Q1 and Q4 (*P* = 0.25). The reported severity of the headaches differentiated two groups of divers; with one group describing headaches as light (grades 1 to 4) and the other group describing them as severe (scores 6 to 10). This last group also represented divers who reported being prone to migraines, thus there could be a link between their sensibility and the severity of the post-saturation headache effects.

The onset of headaches occurred near surface (44%) and after decompression (56%), which coincided with the reduction of the chamber ppO_2_ or the switch to atmospheric 21 kPa ppO_2_. The headache occurrences thus appeared to be synchronized with the changes of inhaled ppO_2._ This is consistent with a reactive cerebral vasodilation due to hypoxia.

#### Post-saturation Fatigue

Most divers reported a feeling of fatigue, lasting up to 10 days after the end of decompression. It is reasonable to expect divers performing intense efforts 8 h a day, for several consecutive weeks, to experience physical fatigue. Several divers involved in night shift dives also mentioned the time required to readjust their circadian rhythms. However, if 71% of the divers reported post-saturation fatigue after their last saturation (question Q8), they also described it as typical and systematic (82% in question Q10). There is no difference between Q8 and Q10 (*P* = 0.5). The feeling of fatigue was presented in the following way:

- The feeling of fatigue was declared to be more physical (82%) than mental (question Q11). This feeling was generally described as a limitation to effort, like becoming breathless when climbing stairs.
- The sense of fatigue remained within moderate levels on a scale from 1 (light) to 10 (severe) [mean ± standard deviation (SD) = 3.4 ± 1.92 in question Q9].
- The overall well-being or mood, graded on a scale from 1 (bad) to 10 (excellent), was not affected (6.18 ± 1.56 in question Q12).
- The capacity to focus or mental alertness, graded on a scale from 1 (bad) to 10 (excellent), was generally unaffected (6.23 ± 1.91 in question Q13). However, one diver mentioned difficulty to concentrate post-saturation.

#### Recovery From Post-saturation Fatigue

All the divers indicated a recovery process and reversible symptoms. However, their post- saturation sense of fatigue could last from 1 to 10 days (4.31 ± 2.92 days in question Q14). One diver reported a recovery in 3 days by comparing his bicycling performance on a regularly used circuit. After returning home, the divers adopted different strategies to manage this feeling of fatigue. Some said they immediately caught up with life and got intensely involved in sport, social life and auxiliary business. Others reported they preferred to take a relaxing week.

An often-depicted relative paleness after decompression was observed on most of the divers. Some divers confirmed that this paleness was noticed by their families after their return home (question Q7, 95%). The divers transferring from the chamber to surface instantly switched from breathing heliox to air. It is conceivable that their bodies may react to the relative drop in oxygen at this point. The transient isobaric counter diffusion and the counter fluxes of helium and nitrogen might have momentarily disturbed alveolar ventilation and reduced oxygen exchanges. The deprivation of sunlight may also contribute to the paleness of the divers.

A study was conducted simultaneously onboard the same vessel in 2016, where hemoglobin and erythropoietin (EPO) levels were measured pre-saturation, immediately post-saturation and 24-h post-saturation (Kiboub et al., 2018a). An increase in EPO was registered over the initial 24-h post-saturation. As EPO regulates erythrocyte production, a post-saturation increase in EPO may counteract the hypoxia perceived after decompression. EPO may thus contribute to the reversible nature of the symptoms observed in this study.

#### Possible Evolution of Saturation Procedures

Considering that most of the saturation procedures used in the offshore industry were designed empirically in the 90s, knowledge obtained via research may contribute to their improvement. The benefit would not primarily be divers’ safety, since saturation diving is already relatively safe (1 decompression sickness per 1,000 exposures) (Petroleum Safety Authority Norway [PTIL], 2018) but rather the divers’ well-being.

During decompression from saturation, the chamber ppO_2_ is linked to the ascent rate (Kot et al., 2015). An experimental saturation dive was performed in Norway in 2004 where a lower chamber ppO_2_ protocol was used. The authors reported a case of decompression sickness and neurological deficit after this dive (Thorsen et al., 2006). The margin for changing ppO_2_ is thus narrow, but there may still be room for improvement. Currently, the ppO_2_ used for the diving mixtures are specified within 60 to 80 kPa. This refers to the US Navy diving manual used in the early 70s, that authorized large excursion distances. These excursion distances have since been restricted to safer values, but the ppO_2_ has remained constant. It is possible that diving mixtures might be redefined according to new experience in the diving industry. The results from the present study may help mitigate some concerns of diving long-terms effects, by showing that some symptoms appear to be related to oxygen acclimatization with reversible short-term effects.

This study has some limitations. When the interviewer was present, questions were answered with 100% compliance. However, in the interviewer’s absence some answers were missed. This is a known limitation; as already shown by other authors in evaluation of other aspects of saturation (Dolan et al., 2016). The data concerning time for recovery after saturation diving were based on the divers’ recollection, and not assessed for the dive after which the questionnaire was completed. Future studies should address the progress of post-saturation recovery concerning symptoms of readjustment to normal life.

## Conclusion

We conclude that post-saturation diving effects on headaches and fatigues include a dimension that is compatible with acclimatization to the higher than normal levels of oxygen experienced in saturation. This assumption is consistent with the hemoglobin concentration changes measured in similar conditions and supported by the subjective evaluation of saturation by the divers as assessed by the questionnaire. These effects appeared reversible post-decompression.

It may be that parts of the alleged long-term effects of saturation diving, developed in 1993 at the Godøysund Conference, are in reality short-termed, reversible effects, associated with oxygen acclimatization.

## Author Contributions

JI, CB, and ØL conception and design of research. JI, FK, and ØL collection of data. JI, FK, IE, and CB analysis and interpretation of data. All participated in the manuscript writing, review, and final approval.

## Conflict of Interest Statement

FK and ØL were employed by TechnipFMC in Norway. The remaining authors declare that the research was conducted in the absence of any commercial or financial relationships that could be construed as a potential conflict of interest.
